# Probing the Y_2_ Receptor on Transmembrane, Intra- and Extra-Cellular Sites for EPR Measurements

**DOI:** 10.3390/molecules25184143

**Published:** 2020-09-10

**Authors:** Jeannette M. Laugwitz, Haleh H. Haeri, Anette Kaiser, Ulrike Krug, Dariush Hinderberger, Annette G. Beck-Sickinger, Peter Schmidt

**Affiliations:** 1 Medical Faculty, Institute of Medical Physics and Biophysics, University of Leipzig, Haertelstasse 16-18, 04107 Leipzig, Germany; jeannette.oettrich@medizin.uni-leipzig.de (J.M.L.); ulrike.krug@medizin.uni-leipzig.de (U.K.); 2 Institute of Chemistry, Martin-Luther-University of Halle-Wittenberg, Von-Danckelmann-Platz 4, 06120 Halle (Saale), Germany; haleh.hashemi-haeri@chemie.uni-halle.de (H.H.H.); dariush.hinderberger@chemie.uni-halle.de (D.H.); 3 Faculty of Life Sciences, Institute of Biochemistry, University of Leipzig, Bruederstrasse 34, 04103 Leipzig, Germany; anette.kaiser@uni-leipzig.de (A.K.); abeck-sickinger@uni-leipzig.de (A.G.B.-S.)

**Keywords:** GPCR, Y2R, EPR, DEER, refolding, nitroxide spin labels, MTSL, IDSL

## Abstract

The function of G protein-coupled receptors is intrinsically linked to their conformational dynamics. In conjugation with site-directed spin labeling, electron paramagnetic resonance (EPR) spectroscopy provides powerful tools to study the highly dynamic conformational states of these proteins. Here, we explored positions for nitroxide spin labeling coupled to single cysteines, introduced at transmembrane, intra- and extra-cellular sites of the human neuropeptide Y2 receptor. Receptor mutants were functionally analyzed in cell culture system, expressed in *Escherichia coli* fermentation with yields of up to 10 mg of purified protein per liter expression medium and functionally reconstituted into a lipid bicelle environment. Successful spin labeling was confirmed by a fluorescence assay and continuous wave EPR measurements. EPR spectra revealed mobile and immobile populations, indicating multiple dynamic conformational states of the receptor. We found that the singly mutated positions by MTSL ((1-oxyl-2,2,5,5-tetramethyl-2,5-dihydro-1H-pyrrol-3-yl) methyl methanesulfonothioate) have a water exposed immobilized conformation as their main conformation, while in case of the IDSL (bis(1-oxyl-2,2,5,5-tetramethyl-3-imidazolin-4-yl) disulfide) labeled positions, the main conformation are mainly of hydrophobic nature. Further, double cysteine mutants were generated and examined for potential applications of distance measurements by double electron–electron resonance (DEER) pulsed EPR technique on the receptor.

## 1. Introduction

G protein-coupled receptors (GPCRs) play a central role in numerous signal transduction pathways across the cell membrane, which are initiated by binding of extracellular ligands to the receptor and subsequent activation of different intracellular signaling pathways. Due to this central role in various biochemical signal transduction cascades, GPCRs are of high pharmacological relevance [1].

The signaling event is no simple “on/off” signal transduction process, but a tightly regulated mechanism inducing multidimensional signaling cascades by receptor coupling to specific intracellular binding partners, like heterotrimeric G-proteins, kinases or arrestins. One major focus in current GPCR research deals with the detailed molecular understanding of the ligand-receptor interaction in terms of structural dynamics features and binding routes. This knowledge may aid the development of highly selective drug molecules that bias one specific signaling pathway [2]. Over the last decade, most structural data for GPCRs was provided by X-ray crystallography [3]. However, the inherent limitation of crystal structures, as they provide only static snapshots of the molecules in a non-membrane environment of artificially stabilized GPCRs [4], are increasingly compensated by other biophysical methods such as cryo-electron microscopy [5,6], nuclear magnetic resonance (NMR) [7,8,9,10,11], or electron paramagnetic resonance (EPR) [12,13] spectroscopy.

EPR has been established as a powerful tool in structural biology and its utilization is based on the presence of unpaired electrons in the system of interest. It is suitable for systems with intrinsically available unpaired electrons (like metalloproteins and metalloenzymes containing transition metals) or requires the incorporation of unpaired electrons to the system of interest, either by spin probing or by spin labeling techniques. The latter, which is also known as site-directed spin labeling (SDSL), has been by far the most common method in biological applications. For this purpose, usually the sulfhydryl group of cysteine residues is attached to nitroxide radicals to form a spin labeled side chain. The cysteine residues either naturally exist or have to be engineered by site-directed mutagenesis into the desired systems. If more than one cysteine is available in the protein, only the desired position is kept and the rest should be eliminated by site directed mutagenesis [14]. The combination of continuous wave (CW)-EPR and SDSL provides information regarding the local environment of the spin label, describing its dynamics on the picosecond timescale and reorientations of the spin-label due to conformational changes of the labeled domain [15,16,17,18]. Utilizing pulsed EPR techniques like double electron–electron resonance (DEER) spectroscopy which is based on the dipolar coupling between two spin centers in the protein, enables probing distance distributions between the spin labels and the possible orientation between them, if the spin label has a preference for specific orientations (the so called orientation selectivity) [19,20,21,22,23]. Additionally, EPR spectroscopy is independent of the system size, which is another advantage of this technique to characterize macromolecular systems [24].

Both, CW- and pulsed EPR techniques have provided information about internal receptor dynamics and conformational plasticity of GPCRs [12,19,25,26]. In CW-EPR the mobility, polarity and conformational distribution of a single nitroxide label at a specific receptor position can be determined [27,28,29,30], while in DEER experiments, the distance distribution between two nitroxide labels of a range from 1.8 to 6 nm is obtained [16]. Using DEER spectroscopy, a recent study of the β2-adrenergic receptor revealed an outward movement of the intracellular end of transmembrane helix (TM) 6 of 14 Å occurs upon binding of a nanobody (a mimic of G protein binding) intracellularly while agonist binding alone causes only minimal changes [12]. In another study, DEER showed that different ligands stabilize distinct receptor conformations of the angiotensin II type 1 receptor [26].

A very successful strategy for GPCR labeling with nitroxide moieties is the introduction of cysteine residues at specific positions in the receptor sequence. However, the challenge in these investigations is to find the optimal position for the cysteine modification on the GPCR molecule that (*i*) is sensitive to the dynamic alterations of the receptor states upon activation or ligand binding events and (*ii*) does not perturb these functional equilibria or the receptor folding in the membrane. Particularly problematic is to find a minimally perturbing site for cysteine mutagenesis on the extracellular receptor side, as these regions are very important for selective agonist binding and most GPCRs exhibit a highly conserved disulfide bridge between the TM3 and the extracellular loop 2 (ECL2). Introduction of any additional non-native cysteine in this region increases the probability of non-native disulfide bridge formation resulting in non-functional receptor molecules. Most studies on GPCRs so far are limited to information from labels on the intracellular ends of the TMs or intracellular loops (ICLs) [12,13,26].

Here, we suggest positions for introducing cysteine residues, which are well distributed over the structure in the human neuropeptide Y type 2 receptor (Y_2_R). The Y_2_R is involved in the regulation of a number of physiological processes including food intake, neuroprotection, and circadian rhythm, and therefore represents an important target for pharmacological intervention [31]. Its natural ligand is the 36 amino acid neuropeptide Y (NPY) [32]. We engineered six mutants, five single site mutants each with one additional cysteine at the intracellular ends of TM3 and TM6, in the middle of TM7, or in the ECL2 and ECL3, and one double mutant with additional cysteines in TM3 and TM7. All mutants were expressed in *Escherichia coli* as inclusion bodies [33], functionally reconstituted into phospholipid bilayer [34], labeled with MTSL or IDSL (see chemical structures in Figure 6a). IDSL is more rigid and can therefore in principle more faithfully report dynamics and distance distributions (via DEER) that are intrinsic to the receptor.

The CW-EPR measurements for all single mutants and for both used spin labels were measured and are discussed. Additionally, DEER measurements were conducted for the double mutant sample, labeled with IDSL.

## 2. Results and Discussion

### 2.1. Selection of Y_2_R Cysteine Mutants

**Figure 1:**
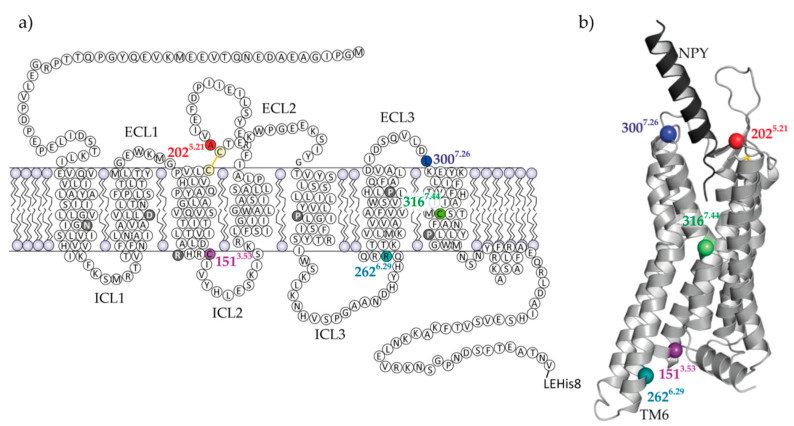
Sequence and structural organization of Y_2_R. The snake plot of the cysteine-deficient Y_2_R variants (**a**) and the structural model [36] of Y_2_R (light grey) in complex with the natural ligand NPY (black) (**b**), illustrate the location of the investigated sites. Mutagenesis sites for introducing cysteines and spin labeling are colored. The artificial C-terminal LEHis8-tag was introduced for purification purposes. The dark grey colored amino acids in the snake plot are highly conserved and constitute the basis for the nomenclature of the amino acids according to Ballesteros and Weinstein [37].

The strategy for site-directed spin labeling using thiol-selective chemistry was designed based on a previously established cysteine deficient Y_2_R mutant, which contains only those two native cysteine residues that form the extracellular disulfide bridge [35]. In addition to a comparable physiological activity of this variant with the wild-type receptor, it is characterized by increased stability which facilitates folding into its native conformation during functional reconstitution [34]. New variants were generated with additional single cysteines at extracellular sites (A202^5.21^C and L300^7.28^C), at the intracellular transmembrane interface (C151^3.53^ and R262^6.29^C), and in helix 7 (C316^7.44^) (Figure 1). Additionally, one cysteine double mutant (C151^3.53^/C316^7.44^) was engineered. In the variants with the mutations C151^3.53^ and C316^7.44^ endogenous cysteines were reintroduced, while in the other three variants the positions of the cysteines were artificial.

Selection of the most optimal spin labeling positions has relied on several criteria. For the extracellular side of the receptor it was most important, that modifications did not affect receptor functionality, ligand binding and/or refolding, in particular disulfide-bridge formation. Alterations at the positions A202^5.21^C and L300^7.28^C in the vicinity of the ligand binding pocket were well tolerated. The new Cys202 residue was introduced as a direct neighbor to the conserved Cys203 and therefore, cannot form a disulfide bridge with Cys203 allowing formation of the conserved disulfide bond to Cys123 in TM3. Although disulfide bridge formation between residue Cys202 and Cys123 cannot be excluded, no effect on spin-labeling or NPY binding could be detected. Other investigated mutation sites within the binding pocket led to intracellular accumulation of the receptor and reduced signaling (Figure S1).

The endogenous cysteine at position C316^7.44^ was identified as best site for effective labeling in the transmembrane region, since the residue points out of the helical bundle facilitating the spin label binding. Furthermore, within this central TM region it was expected that the local environment remains unaltered during protein conformational changes (in terms of interactions to other helices or lipid environment), likely excluding spin-label reorientation and therefore reducing distance distributions in DEER measurements. The second natural cysteine at position C151^3.53^ was defined as a good target because the expected distance to C316^7.44^ is well matched to DEER and is likely to report important conformational changes involving TM3 and TM6. Additionally, because both cysteines are native to the wild type receptor, there were no negative side effects expected on folding of the receptor.

Finally, a labeling site at the intracellular end of helix six was selected with respect to the supposed significance of the large-scale outward movement of TM6 upon activation, which is the most pronounced conformational change typically associated with GPCRs [7,19]. Here, the most suitable position was identified at R262^6.29^ in cell culture experiments. Eight consecutive residues at the intracellular end of TM6 were individually mutated to cysteine in the cysteine-depleted Y_2_R base mutant (Figures S2 and S3). In contrast to other positions, the amino acid exchange at position R262^6.29^C did not interfere with the receptor expression pattern at the cellular surface or with its signalling activity.

**Figure 2:**
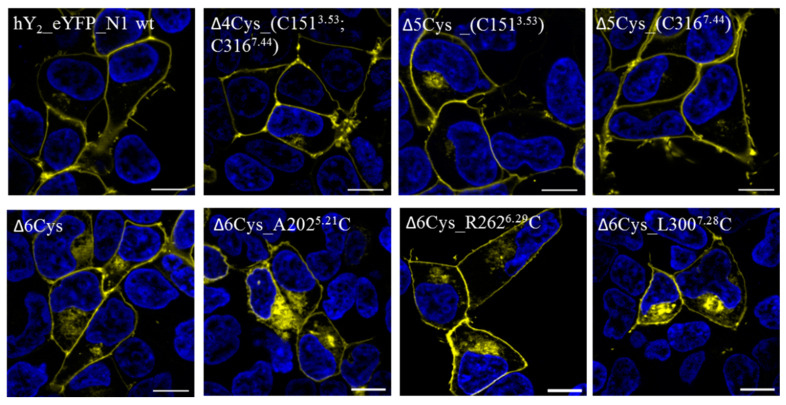
Cellular localization of Y_2_R cysteine variants: The live cell fluorescence images illustrate the cellular expression of eYFP-coupled Y_2_R cysteine mutants compared to the wild type (wt) Y_2_R (hY_2__eYFP_N1 wt). For this purpose, the HEK293 cells were transiently transfected with the corresponding receptor constructs. The cysteine deficient variant (∆6Cys) exhibits a slightly higher intracellular localization compared to the wt receptor, but is still mainly expressed at the cell membrane. Similar expression patterns were observed for the cysteine mutants ∆6Cys_A202^5.21^C, ∆6Cys_R262^6.29^C and ∆6Cys_L300^7.28^C. The cysteine mutants ∆4Cys_ (C151^3.53^; C316^7.44^), ∆5Cys_C151^3.53^ and ∆5Cys_C316^7.44^ are almost completely localized on the cell membrane like the wtY_2_R. Yellow = eYFP, blue = nuclear dye Hoechst 33342. The scale bar corresponds to 10 μm. n ≥ 3.

The influence of the cysteine mutations on the receptor expression and its functionality were examined in cell culture. Receptor localization analysis by live cell fluorescence microscopy in HEK293 cells overexpressing the Y_2_R showed that all of the receptor variants were expressed well and were transported to the plasma membrane (Figure 2). The Δ6Cys variants A202^5.21^C, R262^6.29^C, and L300^7.28^C display slightly altered cellular trafficking, and were partly retained intracellularly in the ER or Golgi, while the wild type Y_2_R was localized exclusively in the plasma membrane.

**Figure 3:**
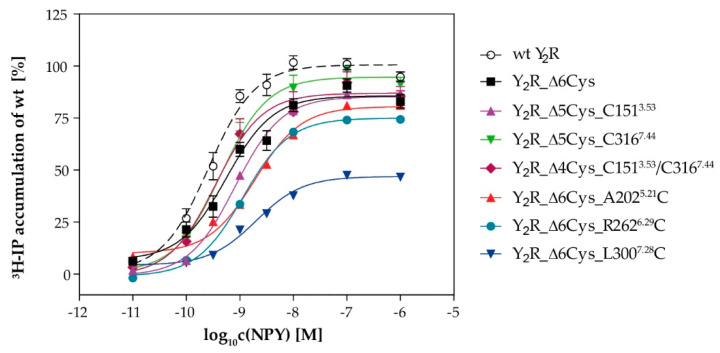
G protein activation of the Y_2_R variants: Receptor activity was determined by inositol phosphate accumulation. COS7 cells were transiently co-transfected with the respective receptor construct and the chimeric G_αΔ4qi6myr_ protein and labeled with ^3^[H]-myo-inositol. All receptor variants were stimulated with NPY for 90 min and accumulated ³H-inositol phosphates were isolated by anion-exchange chromatography. The response was normalized to the wt Y_2_R. Data represent mean ± SEM of n ≥ 2 independent experiments each performed in technical duplicate.

To further verify the functionality of the receptor variants, we measured G protein activation in transfected eukaryotic cells by an inositol phosphate signal transduction assay (Figure 3). We employed a chimeric G protein (G_αΔ6i4myr_, see methods section for details) to redirect the native G_i/o_ signaling to the G_q_ pathway, which can be robustly measured as increase of intracellular inositol phosphates rather than decrease of cellular cAMP. As described previously [35], the cysteine-deficient Y_2_R variant (Δ6Cys) displayed half-maximal activation by NPY at a peptide concentration of 0.6 nM (EC_50_). This was only slightly higher than the wild type receptor which had an EC_50_ of 0.3 nM. Not surprisingly, re-introduction of endogenous cysteines also retained this wild type-like potency (Table 1). Mutation of positions close to the ligand binding pocket at positions A202^5.21^C (ECL2), L300^7.28^C (ECL3) were also tolerated, and displayed EC_50_ values of 2.0 and 2.2 nM, respectively. Similarly, the cysteine mutation at position R262^6.29^C near the G protein binding pocket did not interfere with receptor activity (EC_50_ = 1.1 nM). To verify that this mutation does not interfere with the recognition of the endogenous G_i/o_ protein as well, we employed a second signaling assay that reads out the inhibition of cellular cAMP downstream of the endogenous G_i/o_ proteins. The R262^6.29^C mutation was also tolerated in this setting, and displayed wild-type like EC_50_ values (EC_50_ wt: 11 nM; R262^6.29^C: 77 nM).

Some of the receptor variants, in particular R262^6.29^C and L300^7.28^C, displayed a reduction of the maximal inositol phosphate accumulation. This could be attributed to a reduced surface expression that was also seen in microscopy experiments (cf. Figure 2). The receptor reserve in this assay was low due to the co-transfection of the chimeric G protein. Thus, a reduction of the receptor expression to below ~50% of the wild type value already manifested in reduced E_max_ values, while the receptor remained activatable with good potencies (EC_50_ values). This has already been shown for the Y_1_R in the same assay set-up [38]. We confirmed a reduced receptor expression for the cysteine depleted Y_2_R by determining the number of binding sites (B_max_) and ligand affinity (K_d_) in direct binding assays for the wild type and the cysteine-depleted base mutant. The K_d_ was 23 ± 6 pM and 24 ± 8 pM for wt Y_2_R and Y_2_R _Δ6Cys, respectively, which underlines the high affinity and functionality of the latter construct. However, the B_max_ of the Δ6Cys construct in HEK293 cells was reduced to 44 ± 4% of the wild type receptor, which is also reflected in the small, but statistically significant reduction of the E_max_ in the inositol phosphate signal transduction assay (100% of wt versus 86 ± 2% of Δ6Cys, *p* < 0.001). Furthermore, the reductions in the number of surface receptors will lead to a parallel decrease of the signal window in this signal transduction assay set-up, while the receptor protein is still fully functional. Taken together, these findings indicate that cysteine mutations at these positions are well tolerated and do not interfere with receptor activity.

### 2.2. Protein Expression, Membrane Reconstitution and Spin-Labeling of Y_2_R Cysteine Mutants

**Figure 4:**
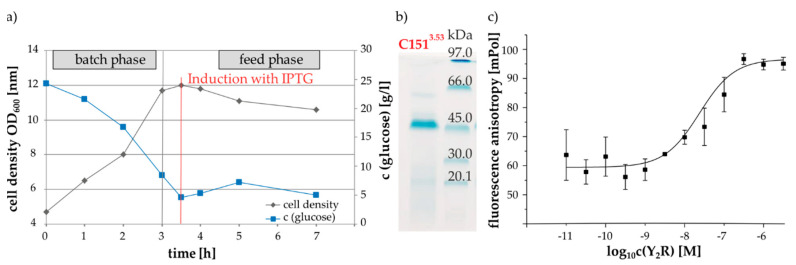
Sample preparation of Y_2_R mutants: (**a**) exemplary curves of the cell density during cultivation and glucose consumption in an *E. coli* fed-batch-fermentation for recombinant expression of the Y_2_R_∆5Cys_C151^3.53^ mutant; (**b**) SDS gel of the solubilized and purified Y_2_R_∆5Cys_C151^3.53^ mutan; (**c**) characterization of ligand binding of the Y_2_R_∆5Cys_C151^3.53^ in bicelles using a fluorescence polarization assay with TAMRA-NPY [34]. Based on the saturation curve a K_D_-value of 25 nM was determined.

The production of large amounts of receptor protein was accomplished by *E. coli* high density fermentation. Figure 4a shows an example of the time courses of cell density and glucose consumption during cultivation of cells expressing the variant Y_2_R_∆5Cys_C151^3.53^. In general, the different Y_2_R-cysteine variants were expressed under the control of a lac-operon and high cell density was generated before induction with IPTG. Afterwards a decrease in optical density was observed, which is due to the formation of toxic hydrophobic receptor domains, enclosed into inclusion bodies and consequently induced cell lysis [39]. Subsequently to the fermentation process, inclusion body purification, receptor solubilization with DTT in sodium dodecyl sulfate (SDS) containing buffer and protein purification by IMAC chromatography were performed, following the descriptions as detailed in the methods section. Exemplarily, the SDS-Gel in Figure 4b illustrates the purified receptor variant Y_2_R_∆5Cys_151^3.53^C in SDS micelles after IMAC purification. The intense blue colored band at 45 kDa was assigned to the receptor mutant and confirmed by mass spectrometry. The expression yields of the different receptor variants ranged between 4 and 13 mg per 1 L fermentation volume and thus reflected lower yields than the Y_2_R_∆6Cys with 20 mg per 1 L fermentation volume as reported before [33]. However, in contrast to the Y_2_R_∆6Cys, the Cys-variants investigated here were not subject to intensive fermentation process optimization [40].

Next, the receptor variants were reconstituted into phospholipid/detergent bicelles composed of 1,2-dimyristol-sn-glycero-3-phosphocholine (DMPC) and 1,2-diheptanoyl-sn-glycero-3-phosphocholine (DHPC-c7), utilizing a tree step in vitro folding protocol that was previously established for the functional reconstitution of Y_2_R_∆6Cys [34]. The ligand-binding ability of the reconstituted Y_2_R variants was confirmed in a fluorescence polarization assay [41]. Exemplary data is shown in Figure 4C. In total, approximately 80% ± 10% of the respective expressed receptor protein was successfully integrated into the membrane system. This corresponds well to the results for the Y_2_R_6Cys [34]. Furthermore, there were only minor differences in the yields and ligand affinities between the individual mutants, which indicate that the cysteine mutations have little influence on the folding process.

**Figure 5:**
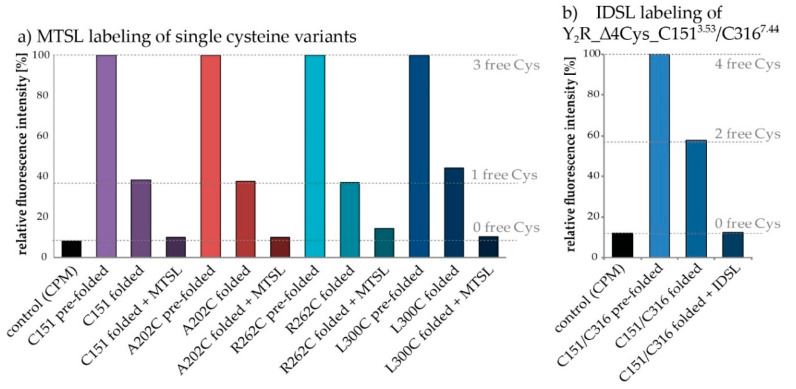
Verification of disulfide bridge formation and nitroxide spin labeling by CPM-Assay: Quantification of free cysteines relies on a thiol-reactive fluorescent probe (CPM), which is essentially non-fluorescent until it reacts with free thiols. The dashed lines indicate the expected relative fluorescence intensity from the number of free cysteines in the respective sample, adjusted to correct for the difference of the signals detected for pre-folded samples (set to 100%) and the control. The pre-folded receptor variants in (**a**) contain three free cysteines, whereas the folded ones have one remaining free Cys. The decrease in fluorescence intensities in all Y_2_R-variants illustrates the completeness of disulfide bridge formation. Furthermore, the spin labeled variants (no free Cys) generate the same weak fluorescence intensity as the control sample, which is an indication of an effective reaction with the spin label. In (**b**) the normalized fluorescence intensity of the cysteine double mutant Y_2_R_∆4Cys_C151^3.53^/C316^7.44^ is shown, containing four free cysteines in its unfolded conformation. The half-maximal fluorescence intensity of the folded receptor (with two cysteines in the disulfide bridge and two free cysteines) and the control-like signal of the IDSL-coupled receptor (two cysteines in the disulfide bridge and two labeled with IDSL) imply successful application of the adapted labeling protocol.

Labeling of the Y_2_R variants with either MTSL or IDSL was achieved by adding a 10-fold excess of the nitroxide spin labels three times to the receptor samples after functional reconstitution with a two-hour incubation time in between each addition. To verify complete disulfide bridge formation and successful spin-labeling free cysteines were labeled with *N*-[4(7-diethylamino-4-methyl-3-coumarinyl) phenyl]maleimide (CPM) and analyzed by fluorescence measurements [42], as shown in Figure 5. The low fluorescence intensity of the control sample (bicelles without receptor) demonstrates that CPM is only weakly fluorescent until reacted with free thiols groups. In contrast high fluorescence intensities were observed for the unfolded receptor variants. To better quantitate the unfolded, folded and labeled receptor, all fluorescence intensities were normalized to the fluorescence intensity of the unfolded form of Y_2_R. As expected, the signals of the folded receptors were reduced by approximately 60% to 70%, which corresponds to a reduction in the number of free cysteines from three to one. Slight deviations from the expected intensities were assigned to the usage of inaccurate protein concentrations caused by weak sample impurities. Moreover, the fluorescence intensities of the labeled receptor samples were comparable to the values of the control, which implies complete spin labeling.

In case of the double mutant an additional labeling step was added directly after the initial dialysis step in which the SDS concentration was reduced and the disulfide bridge was formed but before reconstitution in the lipid membrane. For DEER measurements a nearly 100% labeling efficiency of both positions is essential and it turned out that the additional labeling is necessary for complete saturation of both cysteines with spin labels. In contrast, for the single mutant variants a single labeling step after reconstitution was sufficient. Unfortunately, due to incomplete disulfide bond formation in the initial folding process, the additional labeling step led to minor background labeling of the cysteines forming the disulfide bridge. That can be overcome by correcting the spectra of the double mutants with measurements of background labeling in single mutants, as it has been performed in previous studies [12].

### 2.3. CW-EPR Measurement Analysis of Y_2_R Single Cysteine Mutants

**Figure 6:**
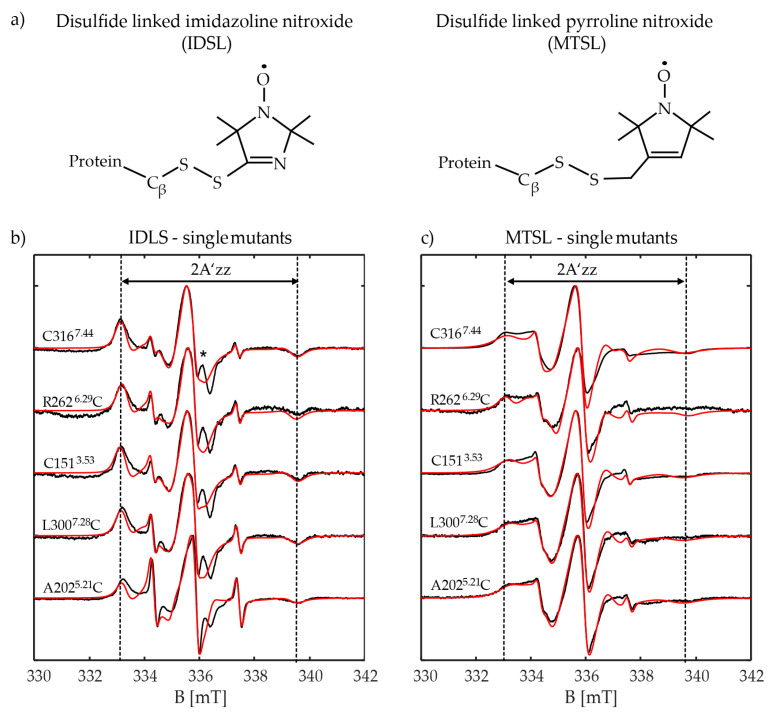
X-band room temperature continuous wave (CW)-EPR measurements of the single cysteine mutants of the Y_2_R: (**a**) chemical structures of IDSL and MTSL are displayed; (**b**) experimental (black) and simulated (red) spectra of Y_2_R single cysteine mutants labeled with IDSL and (**c**) with MTSL. The asterisk indicates the residual fraction of IDSL which is attached to the cysteines of the incompletely formed native disulfide bridge. The distance between the two outermost lines, indicate the apparent hyperfine coupling of the bound spin label to protein (2A’zz).

Room temperature CW-EPR spectra were recorded for all five Y_2_R single cysteine mutants labeled with MTSL or IDSL (Figure 6). The spectral shapes reflect multi dynamic states of the side chains, regardless of whether labeled with MTSL or IDSL. That may arise either from different rotamers of the side chain, each having a different degree of interaction with the environment, or from different conformational states of the protein.The measured spectra were simulated to obtain spectral features like the apparent hyperfine coupling element (A’zz) which is a scale describing the local environment around the spin label (polarity, hydrophobicity, H-bonding) and the rotational correlation time (τ_c_) which represents the label mobility and system dynamics. As a reference measurement, the EPR spectra of two spin labels in buffer were measured and revealed the hydrophobic nature of the IDSL spin label, compared to MTSL (A_iso_ = 42.9 MHz vs 45.4 MHz, respectively). The experimental EPR spectra and their corresponding simulations are given in Figure S4. The values for polarity (A_iso_), the mobility (τ_c_) and the population distribution (Pop) of different conformational states were obtained based on spectral simulations. The results are collected in Table 2.

#### 2.3.1. CW-EPR of Extracellular Positions A202^5.21^C and L300^7.28^C

Simulation of the CW-EPR spectra derived from the MTSL spin labeled ECL positions (A202^5.21^C and L300^7.28^C) of Y_2_R revealed two conformations. The main component, which is populated by about 75%, is in a polar environment. This may be caused by hydrogen bond formation to nearby water molecules as expected for A202^5.21^C, since it is not in the membrane and is more solvent exposed. Additionally, the polar head groups of the nearby lipids from the bicelles may influence the spin label which is a more likely explanation for L300^7.28^C. Both positions showed similar dynamics, slowed down by hydrogen bond formation or spatial hindrances. Using IDSL to spin label the A202^5.21^C-position confirmed the findings by MTSL. The results showed that the water-exposed conformation could now be attributed to an immobile component, having dynamics of about three times slower than the second conformation. This immobile conformation is three times more populated, as observed also by MTSL.

In contrast, for L300^7.28^C IDSL labeling showed different behaviour from MTSL labeling. Although two conformations were also seen, both of these conformations experience a hydrophobic surrounding and one of them has a heavily restricted mobility (~36 ns correlation time). Such a deviation in behavior could be a result of the hydrophobic nature of the IDSL spin label itself, compared to that of MTSL. The rigid behavior of IDSL is based on intra-side chain interactions of the disulfide sulfur atoms with the 3-nitrogen in the imidazoline ring of the spin label [19].

#### 2.3.2. CW-EPR of Intracellular Positions R262^6.29^C and C151^3.53^

Spin labeling of the ICL position R262^6.29^C by MTSL showed two equally populated conformations; an immobile conformation where the spin label is in contact with the polar medium and a flexible conformation located in a hydrophobic environment. Spin labeling by IDSL indicated a heavily restricted and immobile main conformation (80%) which is localized in a hydrophobic environment. A much less populated (18%) and flexible conformation, again of hydrophobic nature, was observed. A similar behaviour could be seen for the other ICL position C151^3.53^ labeled by MTSL. While EPR of the MTSL labeled receptor indicated two conformations with hydrophobic and hydrophilic environments, respectively, the IDSL labeled position mainly adopts a conformation which is of highly hydrophobic nature.

#### 2.3.3. CW-EPR of Transmembrane Position C316^7.44^

For TM position C316^7.44^ the fundamental difference between the two spin labels became apparent. MTSL coupled to C316^7.44^ showed a rather immobile water exposed conformation as its dominant conformation (80%) while IDSL showed a highly populated (>90%) and hindered hydrophobic conformation. According to the model of the Y_2_R [36], this residue points outward from the helical bundle (Figure 1).

#### 2.3.4. CW-EPR of Single Mutants in the Presence of NPY

Addition of NPY did not induce any alterations of either the hyperfine coupling or the rotational dynamics of the protein, irrespective of the spectrometer frequency or applied temperature (measurements performed on 34 GHz (Q-Band) and 100 K are given in Figures S5 and S6). Therefore, this invariant behaviour is not related to the spectral resolution or relaxation effects. That is in agreement with the findings on the β2-adrenergic receptor [12], where addition of the agonist had little effect on the energy landscape of the molecule. Surprisingly, the presence of ligand improves the intensity of the EPR signals (under the same measurement conditions) clearly, which might be caused by increased long term receptor stability in the presence of NPY [43].

#### 2.3.5. CW-EPR of Double Mutants C151^3.53^/C316^7.44^

Double cysteine mutants were prepared once labeled with either MTSL or IDSL. Only the IDSL labeled sample showed changes in its spin quantity (double integration value) and its hyperfine coupling, compared to corresponding single mutants. Simulation of the spectrum of the receptor with no ligand, revealed two conformations, both in a hydrophobic medium (A_iso_ of 43.6 MHz and 42.5 MHz). The main conformation is almost two times more populated (61% vs. 37%) and has about two times slower dynamics, which is in agreement with the intended label positions. In the presence of NPY ligand, the main conformation is more hydrophilic (compared to the one without the ligand), while the second conformation stays in a hydrophobic region (A_iso_ of 44.7 MHz and 40.5 MHz). These findings suggest that in the double mutant sample, the NPY ligand approaches to the receptor from the polar head groups of the lipids from bicelle (Figure S7).

### 2.4. Pulsed EPR Measurement on the Double Mutated C151^3.53^/C316^7.44^

**Figure 7:**
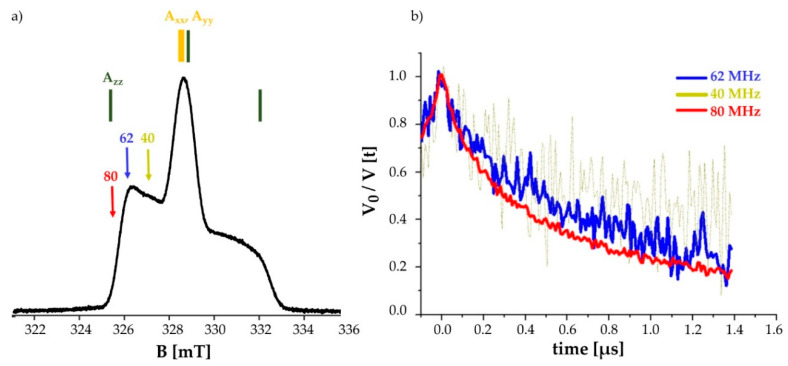
Labeling with IDSL promotes orientation selectivity: (**a**) Echo detected spectrum of the Y_2_R double cysteine mutant C151^3.53^/C316^7.44^ measured at 50 K. At X-band frequencies the z-direction of the ^14^N hyperfine tensor is resolved (shown in green, indicated by A_zz_). The arrows show different positions of the observer frequencies at 40, 62 and 80 MHz. (**b**) DEER time traces of the Y_2_R_Δ4Cys_(C151^3.53^/C316^7.44^) in the presence of NPY ligand at different offsets in equimolar amounts. The poor quality of the signal at an observer frequency of Δν = 40 MHz indicates that the parallel component of the hyperfine tensor has the least contribution in to the DEER time trace.

DEER measurements on the Y_2_R double cysteine mutant were performed using IDSL, since insufficient labeling efficiency of the double mutation using MTSL did not allow for meaningful DEER measurements. As observed from CW-EPR spectra, the imidazole nitroxide IDSL has generated strongly hindered motion of the nitroxide, which suggests the nitroxide spin labels take on specific orientations in the molecular frame of the receptor. Therefore, we performed DEER measurements by putting the observer frequency at three different positions of the echo detected (ED) spectrum. It is well known that the ED spectrum of nitroxides is strongly affected by the anisotropy of the ^14^N hyperfine coupling, which means that by a change in the observer (detection) position, spectra corresponding to different orientations of the spin-label are observed, in case of an orientation preference. Setting the observer frequency at the lower edge of the nitroxide ED signal (Δν = 80 MHz) resulted in the best signal to noise ratio (SNR) while the choice of Δν = 40 MHz produced the poorest SNR. DEER time traces of the three offset positions for the Y_2_R double cysteine mutant with NPY are shown in Figure 7. The measurements on the labeled receptor without the ligand NPY could be performed only at offsets of 62 and 80 MHz, where acceptable SNR were obtained. DEER measurements resulted in signals not only originating from the intended intramolecular distances between spin-labels at C151^3.53^/C316^7.44^, but also between each of these residues and label reacting with the free cysteines of the incompletely formed disulfide bridge, resulting in a multi-spin system. Therefore, we refrain from further analysis of DEER data.

**Figure 8:**
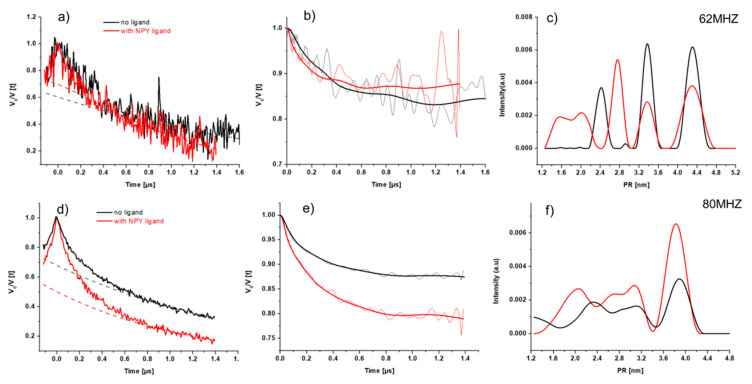
DEER measurements of the Y_2_R at different offsets in the presence and absence of ligand NPY at 50K. From left to right are given DEER raw data (left) with applied background lines dashed lines), background corrected DEER time traces with their fit (solid line) (in center) and the corresponding distance distributions (right). The upper panel shows the DEER data measured at 62 MHz (**a**–**c**). The DEER data at 80 MHz offset are given in the lower panel (**d**–**f**).

Nonetheless, we observed differences not only due to the orientation preference of the spin labels, but also various states of receptor with and without the presence of its ligand NPY. The corresponding DEER time traces are given in Figure 8 and Figure S8. In an initial estimation, the presence of NPY promotes shorter individual distances, confirmed by using the validation tool described in methods (cf. Section 4.8.2 and Figure S9) we obtain a mean distance ~3.7–3.9 nm for the complex of receptor and ligand. For the receptor, this value increases to 4.5 nm. Whether this is a true structural effect, or only due to additional labeling of cysteines supposed to be involved in forming disulfide bonds, which is different in the apo- and the NPY -bound state (cf. Section 4.8.1), cannot be answered unambiguously. A comprehensive picture of the resulting distance distribution will be subject to further extensive studies. However, based on the presented study, numerous distances between diverse receptor sites and from different receptor states can be obtained, which, together with the DEER data, will facilitate modelling of the distance distributions.

## 3. Conclusions

The understanding of the dynamic nature of GPCRs and changes in their conformational equilibrium during the signal transduction pathways through the membrane is one prerequisite for the development of efficient pharmaceuticals. EPR spectroscopy based on nitroxide spin labels coupled to cysteines introduced into the receptor can provide information about intra- and intermolecular distances, site-specific mobility and polarity, as well as conformational distributions. To gain a more complete picture, it is helpful to introduce labels at different receptor sites, which are sensitive to conformational changes. While labels at the intracellular side of GPCRs have provided important findings about structural changes of receptors upon activation, information from labels at the TM region or the extracellular side are rare [12,26]. TM regions are often difficult to label, because of the hydrophobicity of the 7-TM helical bundle and its surrounding membrane. Introducing cysteine residues at the extracellular side often leads to misfolded and non-functional receptor molecules.

Here, we identified and characterized five positions in Y_2_R for introducing cysteines and nitroxide spin labeling which are well distributed over the important receptor regions and well tolerated for receptor folding. Two of these positions are on the extracellular side, two on the intracellular side, and one in the TM region. All five Y_2_R mutants were expressed in *E. coli* and functionally reconstituted into the membrane environment with milligram amounts of proteins. A protocol for efficient spin labeling is presented and CW-EPR spectra of the Y_2_R mutants labeled with either MTSL or IDSL were recorded, displaying differences in the mobility of the labels at the different sites.

Finally, double cysteine mutants were generated and the efficiency of DEER measurements was examined for the future applications in structural studies of Y_2_R. However, methods to achieve 100% labeling while retaining the desired disulfide bonds are needed in order to eliminate interference multi-spin effects. Nevertheless, especially labeling the positions on the extracellular receptor side overcomes limitations in previous studies and holds the promise of determining intermolecular distances between peptide ligands and the receptor using spin labeled peptides. That methodology could be applicable to peptide-binding receptors generally.

## 4. Materials and Methods

### 4.1. Materials

DMPC and DHPC-c7 for bicelle preparation were obtained from Avanti Polar Lipids, Alabaster, AL, USA. The nitroxide spin labels MTSL was purchased from Santa Cruz Biotechnology (Cat# sc-208677) and IDSL from ENZO (Cat# ALX-430-120).

### 4.2. Peptide Synthesis

The ligand NPY (YPSKPDNPGEDAPAEDLARYYSALRHYINLITRQRY–NH2) was obtained from automated Fmoc/tertButyl solid-state peptide synthesis (SPPS) in 15 μM scale on Rink amide resin, cleaved from the resin and purified to >95% as described previously [36]. For application in binding assays, a fluorescent peptide variant was generated. To this end, 5/6-carboxy-tetramethylrhodamine (TAMRA) was attached to the free *N*-terminus on resin after automated SPPS, by using 2 equivalents each of the fluorescence dye (free acid), *O*-(7-azabenzotriazole-1-yl)-1,1,3,3-tetramethyluronium-hexafluorophosphate (HATU) and *N*,*N*-diisopropylethylamine (DIPEA) in 300 μL dimethylformamide (DMF) for 3 h at room temperature.

### 4.3. Generation of Plasmids

Cysteines were introduced as single mutations (C151^3.53^, A202^5.21^C, R262^6.29^C, L300^7.28^C and C316^7.44^) or pairwise as double mutations (C151^3.53^/C316^7.44^) into a cysteine-depleted Y_2_R base mutant (Y_2_R_C58^1.40^A_C103^2.57^S_C151^3.53^S_C272^6.39^A_C316^7.44^A_C342^7.70^A; in either eukaryotic expression vector eYFP_N1 or vector for prokaryotic expression 8xHis_pET41b [35] by site-directed mutagenesis (QuikChange^®^, Stratagene, Agilent, Santa Clara, CA, USA). Sequence accuracy was confirmed by Sanger DNA sequencing. Residue nomenclature follows Ballesteros and Weinstein [37].

### 4.4. Cell Culture Studies

Cell culture materials were purchased from PAA (Pasching, Austria) and plastics from TPP (Trasadingen, Switzerland). HEK293 cells were grown in Dulbecco’s modified Eagle’s medium/Ham’s F12 nutrient mix (DMEM/Ham’s F12) supplemented with 15% (*v*/*v*) fetal calf serum (FCS). COS-7 cells were cultivated in DMEM + 10% FCS. All cells were kept as monolayers at 37 °C, 5% CO_2_ in humidified atmosphere.

#### 4.4.1. Live-Cell Fluorescence Microscopy

Correct expression and subcellular localization of the receptor mutants was investigated in HEK293 cells. HEK293 cells were seeded onto μ-slides (IBIDItreat, Martinsried, Germany). At 70% confluence, the cells were transfected with 500 ng plasmid DNA encoding a genetic fusion of the indicated Y_2_R variant with eYFP (eYFP_N1 vector) using Lipofectamine2000 (Invitrogen, Carlsbad, CA, USA) according to the manufacturer’s instructions. 16–24 h post transfection, nuclei were stained with Hoechst33342, and the cells were imaged using an Axiovert Observer Z1 microscope with automatic light exposure (with Apotome, Plan-Apochromat 63x/1.40 Oil DIC objective, filter sets 02 (365/420), 46(500/535); Carl Zeiss, Jena, Germany).

#### 4.4.2. Signal Transduction via Chimeric G_αΔ6qi4myr_

Activity of the Y_2_R mutants was tested in the inositol phosphate signal transduction experiments. In this assay set-up, a chimeric G_αΔ6qi4myr_ protein is co-transfected to redirect the endogenous Gi/o signaling to the phospholipid C pathway [44,45]. A detailed protocol is described elsewhere [35]. Briefly, COS7 cells were transiently transfected with plasmids encoding the indicated Y_2_R variant (eYFP_N1 vector) and the chimeric G protein. The cells were labeled with 2 µCi/mL ³H-myo-inositol (Perkin Elmer, Waltham, MA, USA) overnight, stimulated with different concentration of peptide for 90 min, and ³H-inositol phosphates were isolated from the cell lysates by anion-exchange chromatography and measured by liquid scintillation counting. Concentration–response curves are the mean of >2 independent experiments performed in technical duplicate and were analyzed with GraphPad Prism 5.03 (GraphPad Software, San Diego, CA, USA) using a three-parameter sigmoidal fit with fixed Hill coefficient (n_H_ = 1).

#### 4.4.3. Signal Transduction via Endogenous Gα_i/o_

Signal transduction of selected Y2R variants with single cysteines introduced to intracellular regions of the receptor was also assayed in a reporter gene assay based on endogenous Gi/o (OneGloTM luciferase reporter gene assay, Promega, Madison, WI, USA). Plasmids encoding the indicated receptor variant (4 μg, eYFP_N1 vector) and luciferase reporter pGL4.29[luc2P/CRE/Hygro] (4 μg) were cotransfected into 70% confluent HEK293 cells in 6-well plates using Lipofectamine2000 (Life Technologies, Thermo Fisher Scientific, Carlsbad, CA, USA) lipofection reagent according to manufacturer’s instructions. One day post transfection, the cells were re-seeded onto poly-D-lysine coated 96-well plates (white, clear bottom; 125,000 cells/well), and grown for another day. Prior to stimulation, the cells were starved for 1 h in serum-free medium, and stimulated with varying NPY concentrations (10-11 M – 10-5 M) in serum-free medium for 2 h. The peptide solutions were removed and the cells were incubated with 1 μM forskolin (Sigma-Aldrich, St. Louis, MO, USA) for 2 h. The cells were washed once, 30 μL serum-free medium/well was added and the cells were equilibrated to room temperature for 10 min. Subsequently, 30 μL OneGlo reagent/well (room temperature) was added, and incubated 5 min in the dark before measuring luminescence in a plate reader (Tecan Infinite 200, Tecan, Männedorf, Switzerland). Concentration-response curves are the mean of >2 independent experiments performed in technical triplicate and were analyzed with GraphPad Prism 5.03 (GraphPad Software, San Diego, CA, USA) using a three-parameter sigmoidal fit with fixed Hill coefficient (nH = 1).

#### 4.4.4. Radioligand Binding Assays

The affinity (Kd) and number of binding sites (Bmax) of selected Y_2_R variants was determined by radioligand binding assays using ^125^I-PYY (Perkin Elmer, Waltham, MA, USA) as described [46].

### 4.5. Y_2_R Expression and Purification

Y_2_R receptor mutants, C-terminally flanked with a poly-8-His-tag, were expressed in *E.coli Rosetta^TM^ (DH3)pLysS* or *NiCo21(DH3)* strains as inclusion bodies during a fed-batch fermentation run in defined minimal salt medium as described previously [33]. Purification of inclusion bodies, protein solubilization in SDS and DTT containing buffer and the His-tag based IMAC purification of the unfolded receptor proteins, were done according to the well-established standard protocol [47].

### 4.6. In Vitro Folding and Nitroxide Spin Labeling of the Y_2_R-Variants

The refolding of the Y_2_R variants into a functional state was performed according to a three-step folding protocol as described before [34]. Briefly, in the first step the extracellular disulfide bridge is formed using a glutathione redox shuffling system within a dialysis process of the purified receptor against degassed buffer, containing 50 mM sodium phosphate at pH 8.5, 1 mM SDS, 1 mM EDTA, 0.2 mM reduced glutathione and 0.1 mM oxidized glutathione at room temperature for 72 h using dialysis tubing with 6–8 kDa cut-off. Next, fresh bicelles consisting of DMPC and DHPC in a molar ratio of 1: 4 DMPC: DHPC (q-value: 0.25) were prepared in 50 mM sodium phosphate buffer, pH 7. The second in vitro folding step comprised the reconstitution of the receptor sample into the prepared DMPC/DHPC-bicelles in a molar ratio of 1:180 Y_2_R: DMPC, whereby both solutions were carefully mixed and subsequently integrated into the membrane system during three cycles of fast-temperature changes from 42 °C to 0 °C, with an incubation time of 20 min each. In step three, the excess of the SDS and DHPC were removed to concentrate the receptor in large, non-isotropic bicelles (q > 10) by using 50 mg/mL BioBeadsSM2. The BioBeads were added at least twice and incubated at room temperature while shaking in the dark until the solution became turbid. Finally, the BioBeads were removed and the sample intensively washed three times by centrifugation and re-suspension of the pellet with 1 mL 50 mM sodium phosphate, pH 7, to purify it from remaining glutathione and EDTA. Nitroxide spin labeling was achieved by adding MTSL to the respective receptor sample in three steps with a 10-fold molecular excess in each step. The first two steps were followed by an incubation period of two hours at room temperature and after the third step the samples were incubated overnight at 4 °C. Finally, the reconstituted receptor was pelleted by centrifugation (8 min, 4 °C, 21,500× *g*) and re-suspended in sodium phosphate buffer pH 7 eight times to ensured complete removal of free MTSL. The receptor concentration was measured by UV-vis spectroscopy at a NanoDrop system at 280 nm absorption, after dilution of the sample in a 5 or 10-fold volume of 50 mM sodium phosphate buffer, pH 8, containing 15 mM SDS.

### 4.7. Flourescence Spectroscopy

The assessment of disulfide bridge formation and effective spin labeling was performed by the coupling of a thiol-specific fluorochrome *N*-(4(7-diethylamino-4-methyl-3-coumarinyl) phenyl)maleimide (CPM) to free cysteines. CPM was dissolved in DMSO to a final stock concentration of 4 mg/mL. The CPM stock solution was diluted 40-fold with 50 mM sodium phosphate buffer pH 7 to a concentration of 0.1 mg/mL just prior to the experiment. From the receptor samples, collected at certain time points during sample preparation, a total amount of 10 µg protein was re-suspended in 15 mM SDS containing 50 mM sodium phosphate buffer, pH 7 to a final volume of 720 µL. The receptor samples were subsequently mixed with 60 µL of the prepared CPM solution and incubated at room temperature in the dark for 15 min. Fluorescence intensities were determined on FluoroMax-2 (JOBIN YVON) in a 10 mm quartz cuvette with an excitation wavelength of 387 nm, scanning emission wavelength from 450 to 500 nm, and an integration time of 0.5 s. All samples were scanned three times at 20 °C.

### 4.8. EPR-Spectroscopy

#### 4.8.1. CW-EPR Spectroscopy

The final sample preparation was performed as follows: (1) Receptor samples in the apo-state were stored as pellets at −20 °C and directly before the measurements re-suspended in 50 mM sodium phosphate pH 7 to reach a final concentration of 200–250 µM. (2) To prepare samples in the ligand-bound state, receptor pellets were re-suspended with a solution containing the native ligand NPY in a molar ratio of 1:2 (Y_2_R: NPY) and incubated overnight at 4 °C in the dark. Afterwards the samples were pelleted, frozen and dissolved like the receptor samples in the apo-state.

X-Band (~9.4 GHz) CW-EPR measurements were performed on a Magnettech MiniScope MS400 benchtop spectrometer (Magnettech, Berlin, Germany) at room temperature. EPR spectra were recorded using a microwave power of 3.16 mW, 100 kHz modulation frequency, a modulation amplitude of 0.2 mT and 4096 points. The final spectra were accumulated from 10 scans, each acquired in 60 s.

Low temperature (100 K) Q-band CW-EPR (34 GHz) measurements were conducted on a Bruker EMX-plusQ spectrometer, using an ER5106QT resonator. Microwave power was set to 2.3 mW and a modulation amplitude of 0.15 mT was used during measurements. The modulation frequency was set to 100 kHz. A Sumitomo cryo compressor-F70 was used for cooling together with a Mercury iTC (Oxford Instruments) to control the temperature.

EPR spectra were simulated based on the spin Hamiltonian using the easyspin software package (release 5.2.25) [48]. Natural abundancies of the nuclei were used throughout the simulations. The principal values of the g-tensor were chosen according to the values reported for hydrophobic media [49,50,51] as the follows: g_zz_ = 2.0023, g_yy_ = 2.0062 and g_xx_ = 2.0089.

A three-component system was considered for simulations, containing immobile and mobile components and free (non-bound) spin label. Therefore, we could estimate the hyperfine couplings, rotational correlation times and population of each of these components, using the double integral value for the latter. The errors of the simulations were calculated based on root-mean square difference (RMSD) between experimental and simulated spectra. For MTSL, the error was in the range of 9–12%. As expected, in case of IDSL spectral simulation, a bigger error value was obtained (15–20%) since the residual fractions (shown by an asterisk in Figure 6) of the IDSL were not considered in the simulation. None of these error values promotes significant changes on the final isotropic hyperfine values and therefore the spin label moiety.

The labeling efficiency of all mutants were obtained by comparing their double integral value with that of a free spin label in buffer with a known concentration of 200 µM and by considering the measurement conditions, such as microwave power, modulation amplitude and receiver gain. Labeling efficiencies for MTSL single mutants were found as A202^5.21^ (81%), L300^7.26^C (100%), R262^6.29^ (81%), C151^3.53^ (100%) and C316^7.44^ (80%). The values for IDSL single mutants were estimated as A202^5.21^ (60%), L300^7.26^C (43%), R262^6.29^ (24%), C151^3.53^ (31%) and C316^7.44^ (44%). Using the same method and considering that indeed in the double mutants are four spin centres in the sample, we found labeling efficiencies of 100% and 25% for the sample with and without NPY, respectively.

The rotational correlation times were assessed using an isotropic model [52] in which, the $D_{i\;(i\;=\;\left(x,y,z\right))}$ are the principal elements of the diffusion tensor.

$$\tau_{c}=\left(\frac{1}{6\sqrt[{3}]{D_{x}D_{y}D_{z}}}\right)$$

#### 4.8.2. DEER Experiments

For DEER experiments, a 3-step in-vitro folding protocol for enhanced nitroxide spin labeling was established. After the first dialysis step of disulfide bridge formation, the sample was dialyzed against the same buffer conditions for 24 h, excluding the glutathione-shuffling system to avoid cross-reactions during the spin labeling process. Subsequently, the respective receptor sample was incubated three times with a 10-fold molar excess of IDSL for 2 h in the first two incubation periods and finally over night at room temperature with rotation, always in the absence of light. Thereafter sample preparation followed the standard protocol, except that a higher receptor: DMPC ratio of 1:400 was used. Furthermore, after the final reconstitution steps, the Y_2_R_Δ4Cys_(C151^3.53^/C316^7.44^) was additionally incubated with the nitroxide spin label overnight at 4 °C to enhance the labeling efficacy. To investigate the ligand-bound state, NPY was added to the receptor solution in a 2-fold molar excess in the overnight IDSL-labeling step. Subsequently, the samples were pelleted and re-suspended eight times as described in Section 4.6 to remove free spin label as well as free NPY and were concentrated to 250 µM in 50 mM NaP, pH 7. The NPY-bound receptor was then incubated one more time for 15 min with NPY at room temperature. Finally, the samples were mixed with 20% glycerol, flash frozen in liquid nitrogen in 3 mm (outer diameter) EPR quartz tubes (Qsil, Germany) and stored at −80 °C.

X-band (~9.4 GHz) pulsed EPR measurements were performed on a Bruker Elexsys E580 spectrometer equipped with a 3 mm Flexline split-ring resonator (ER4118X-MS3-Bruker Biospin GmbH, Karlsruhe, Germany). The resonator could be over-coupled to Q~100. The pulses were amplified by a 1 kW pulsed travelling wave tube (TWT) amplifier (Applied system Engineering, TX, US, Model117). Echo detected field sweep (Electron Spin Echo-ESE), T_2_ relaxation measurements and DEER measurements were recorded at 50 K which is achieved by a closed cycle cryostat (ARS-4WH, www.arscryo.com) and a lake Shore Cryotronics temperature controller (Westerville, OH, USA). ESE spectra were obtained with the conventional two-pulse echo sequence π/2-t-π (t = 176 ns) in which the echo intensity is monitored as a function of the magnetic field. A conventional four pulsed DEER sequence is used for the measurements; π/2(observer) -t_1_-π(ν_observer_)-t-π(ν_pump_)-(t_1_ + t_2_ − t)-π(ν_observer_)-t_2_ – echo. The length of both π and π/2 pulses for the observer sequence were 32 ns to assure equal excitation bandwidths. The length of the pump pulse was 12 ns. The initial t_1_ was set to 180 ns. To supress the nuclear modulation effects, they were averaged in 8 ns steps. Time traces were recorded for a t_2_ of 1.5 μs. The pump pulse was set to the maximum of the nitroxide field sweep spectrum, while the observer frequency was set on the low field shoulder at three different position (offset frequency (Δν) = 40, 62 and 80 MHz) to examine the orientation selectivity induced by the spin label, IDSL. Each measurement took about 24 h corresponding to 1800–2000 scans for each time trace.

DEER time traces and error analysis of the obtained distance distributions were processed with the MATLAB-based (release: R2016a) DEER Analysis package developed by Jeschke and co-workers (release: 2016) [53]. The background of the original DEER time traces was corrected using stretched exponential functions with homogeneous dimensions. The effect of uncertainty of background dimensionality (11 trials) and background starting time (21 trials) were examined using the validation tool, as implemented in DEERAnalysis, resulted in a background dimensionality of 3.0 for two samples. The Tikhonov regularization was used to extract distance distributions from the background corrected time with a regularization parameter of α = 100 for all measurements. To account for the contribution of the multi-spin effects in the distance distributions and suppression of the artefacts, the ghost suppression option for four spins was used throughout the data analysis.

### 4.9. Analysis Tools

GPCR structure was visualized using the PyMOL software (PymolTM Educational Product, copyright © 2010 Schodinger, LLC).

## Figures and Tables

**Table 1:** Compilation of the determined EC_50_, pEC_50_ and E_max_ values from the concentration-response curves of the Y_2_R variants.

	EC_50_ [nM]	pEC_50_ ± SEM	E_max_ ± SE [%]
Y_2_R_eYFP_N1 (wt)	0.3	9.59 ± 0.07	100
Y_2_R_Δ6Cys	0.6	9.25 ± 0.09	86 ± 2
Y_2_R_Δ4Cys_C151^3.53^/C316^7.44^	0.4	9.45 ± 0.15	87 ± 3
Y_2_R_Δ5Cys_C151^3.53^	0.8	9.09 ± 0.10	85 ± 3
Y_2_R_Δ5Cys_C316^7.44^	0.4	9.45 ± 0.15	87 ± 3
Y_2_R_Δ6Cys_A202^5.21^C	2.2	8.66 ± 0.18	81 ± 5
Y_2_R_Δ6Cys_R262^6.29^C	1.1	8.94 ± 0.17	75 ± 4
Y_2_R_Δ6Cys_L300^7.28^C	2.0	8.70 ± 0.10	47 ± 2

**Table 2:** EPR spectral simulation data of single spin labeled Y_2_R by MTSL and IDSL at extracellular (ECL), intracellular (ICL) and transmembrane (TM) positions. The hyperfine tensor and isotropic hyperfine couplings and the rotational correlation times are given in MHz and nanoseconds, respectively.

	MTSL		IDSL	
	Conformation1	Conformation2	Conformation1	Conformation2
	A202^5.21^C (ECL)			
[A_x_, A_y_, A_z_] A_iso_	[18.5, 18.3, 108.0] 48.3	[18.5, 15.3, 95.7] 43.2	[18.5, 18.3, 98.3] 45.0	[18.5, 15.3, 88.7] 40.8
τ_c_	5.5	4.7	9.9	2.9
Pop%	75.2	23.6	63.5	30.7
	L300^7.26^C (ECL)			
[A_x_, A_y_, A_z_] A_iso_	[18.5, 18.3, 108.0] 48.3	[19.5, 15.3, 95.7] 43.5	[18.5, 14.3, 95.0] 42.6	[15.5, 15.3, 93.7] 41.5
τ_c_	5.1	4.4	35.9	4.7
Pop%	77.7	21.5	64.4	34.0
	R262^6.29^C (ICL)			
[A_x_, A_y_, A_z_] A_iso_	[16.5, 18.3, 106.0] 46.9	[16.5, 16.3, 95.7] 42.8	[14.5, 15.3, 93.0] 40.9	[15.3, 15.3, 90.7] 40.4
τ_c_	15.6	6.6	36.3	4.3
Pop%	49.6	49.3	80.7	18.2
	C151^3.53^ (ICL)			
[A_x_, A_y_, A_z_] A_iso_	[19.5, 15.3, 106.8] 47.2	[19.5, 15.3, 95.7] 43.5	[19.5, 14.3, 95.8] 43.2	[18.5, 18.3, 90.7] 42.5
τ_c_	6.0	2.6	15.3	4.9
Pop%	85.3	14.2	87.2	11.5
	C316^7.44^ (TM)			
[A_x_, A_y_, A_z_] A_iso_	[19.5, 14.3, 111.0] 48.3	[19.5, 15.3, 95.7] 43.5	[19.5, 14.3, 95.0] 42.9	[18.5, 15.3, 95.7] 43.2
τ_c_	5.7	1.1	15.3	4.7
Pop%	93.7	6.2	82.2	16.7

## Supplementary Materials

The following are available online, Figure S1: Investigation of further extracellular cysteine mutations to identify possibly spin labeling sites. Figure S2: Positional scan to identify suitable positions for cysteine introduction. Figure S3: Detailed characterization of Y2R Cys-dpl + R6.29C/R6.30C. Figure S4: Room temperature X-band EPR spectra of the used spin labels in NaP buffer. Figure S5 (a–c): Room temperature X-band EPR spectra of the single mutants labeled with MTSL and IDSL in the presence and absence of NPY ligand for (a) intracellular, (b) extracellular and (c) transmembrane positions. Figure S6: Overlaid low temperature (100K) Q-band CW-EPR spectra of the two extracellular and 22 transmembrane positions and labeled with MTSL in the presence and absence of the NPY ligand. Figure S7: Room temperature CW-EPR experimental (black) and simulated (red) spectra of double mutants, 24 labeled with IDSL. With and without the NPY ligand. Figure S8: DEER measurements of the Y2 receptor at different offsets in the presence of NPY at 40MHz. Figure S9: The influence of uncertainties of the background correction and dimensionality on the distance 27 distributions in the presence and absence of NPY ligand at different offsets.
