# Supplementary material for: Probing the Y2 Receptor on Transmembrane, Intra- and Extra-Cellular Sites for EPR Measurements
Source: Molecules. 2020 Sep 10;25(18):4143. doi: 10.3390/molecules25184143 (PMC7571237; doi:10.3390/molecules25184143)
Supplement: Supplementary file 1 [file molecules-25-04143-s001.pdf]

molecules

SUPPLEMENTARY MATERIALS

Probing the Y2 Receptor on Transmembrane, Intra- and Extra-Cellular Sites for EPR Measurements

Jeannette M. Laugwitz[1], Haleh H. Haeri[2], Anette Kaiser[3], Ulrike Krug[1], Dariush Hinderberger[2], Annette G. Beck-Sickinger[3], Peter Schmidt[1*]

[1] University of Leipzig, Medical Faculty, Institute of Medical Physics and Biophysics; Haertelstasse 16-18, Leipzig, 04107 Germany
[2] Martin-Luther-University of Halle-Wittenberg, Institute of Chemistry; Von-Danckelmann-Platz 4, Halle (Saale), 06120 Germany;
[3] University of Leipzig, Faculty of Life Sciences, Institute of Biochemistry, Bruederstrasse 34, Leipzig, 04103 Germany
* Correspondence: peter.schmidt@medizin.uni-leipzig.de

SUPPLEMENTARY MATERIALS CONTENT

Figure S1: Investigation of further extracellular cysteine mutations to identify possibly spin labeling sites.

Figure 2S: Positional scan to identify suitable positions for cysteine introduction.

Figure S3: Detailed characterization of Y2R Cys-dpl + R6.29C/R6.30C

Figure S4: Room temperature X-band EPR spectra of the used spin labels in NaP buffer

Figure S5 (a-c): Room temperature X-band EPR spectra of the single mutants labeled with MTSL and IDSL in the presence and absence of NPY ligand for (a) intracellular, (b) extracellular and (c) transmembrane positions.

Figure S6: Overlaid low temperature (100K) Q-band CW-EPR spectra of the two extracellular and transmembrane positions and labeled with MTSL in the presence and absence of the NPY ligand

Figure S7: Room temperature CW-EPR experimental (black) and simulated (red) spectra of double mutants, labeled with IDSL. With and without the NPY ligand.

Figure S8: DEER measurements of the Y2 receptor at different offsets in the presence of NPY at 40MHz

Figure S9: The influence of uncertainties of the background correction and dimensionality on the distance distributions in the presence and absence of NPY ligand at different offsets.

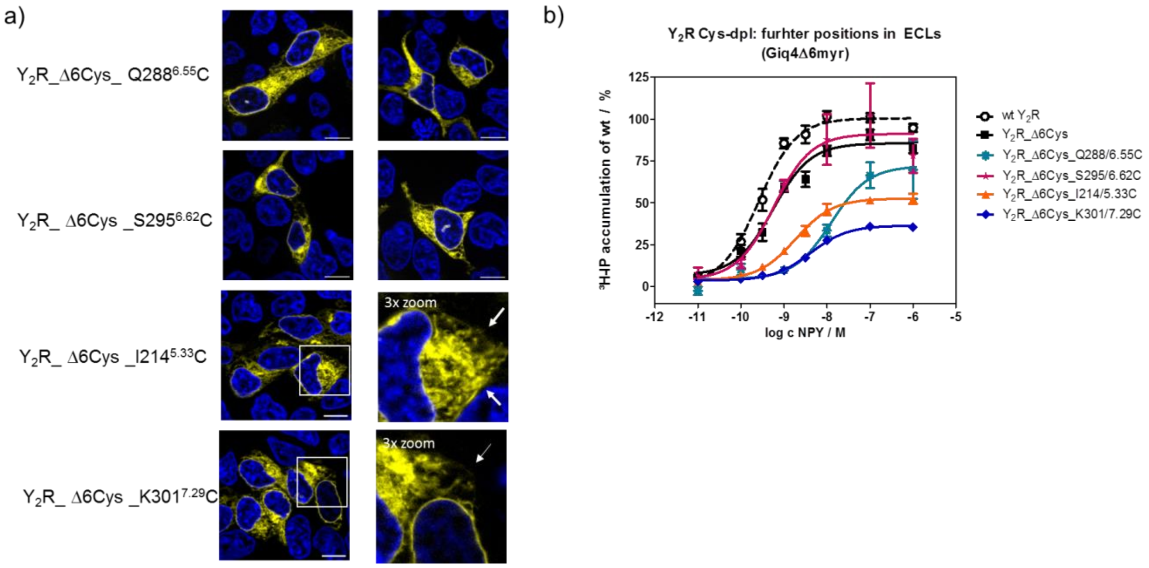

Figure S1: Investigation of further extracellular cysteine mutations to identify possibly spin labeling sites. a) Live cell fluorescence images illustrate the cellular expression of eYFP-coupled Y_2R cysteine mutants. For this purpose, the HEK293 cells were transiently transfected with the corresponding receptor constructs. The cysteine mutants were almost completely accumulated in intracellular compartments and were not transported to the cell membrane, indicating possibly structural deficits. Yellow = eYFP, blue = nuclear dye Hoechst 33342. The scale bar corresponds to 10 μm. n ≥ 3. **b)** G protein activation of the alternative Y_2R cycteine variants illustrate decreased receptor signalling. Receptor activity was determined by inositol phosphate accumulation. COS7 cells were transiently co-transfected with the respective receptor construct and the chimeric $G_{\alpha\Delta4qi6myr}$ protein and labeled with ^{3}[H]-myo-inositol. All receptor variants were stimulated with NPY for 90 min and accumulated ^{3}H-inositol phophates were isolated by anion-exchange chromatography. The response was normalized to the wt Y_2R. Data represent mean ± SEM of n ≥ 2 independent experiments each performed in technical duplicate.

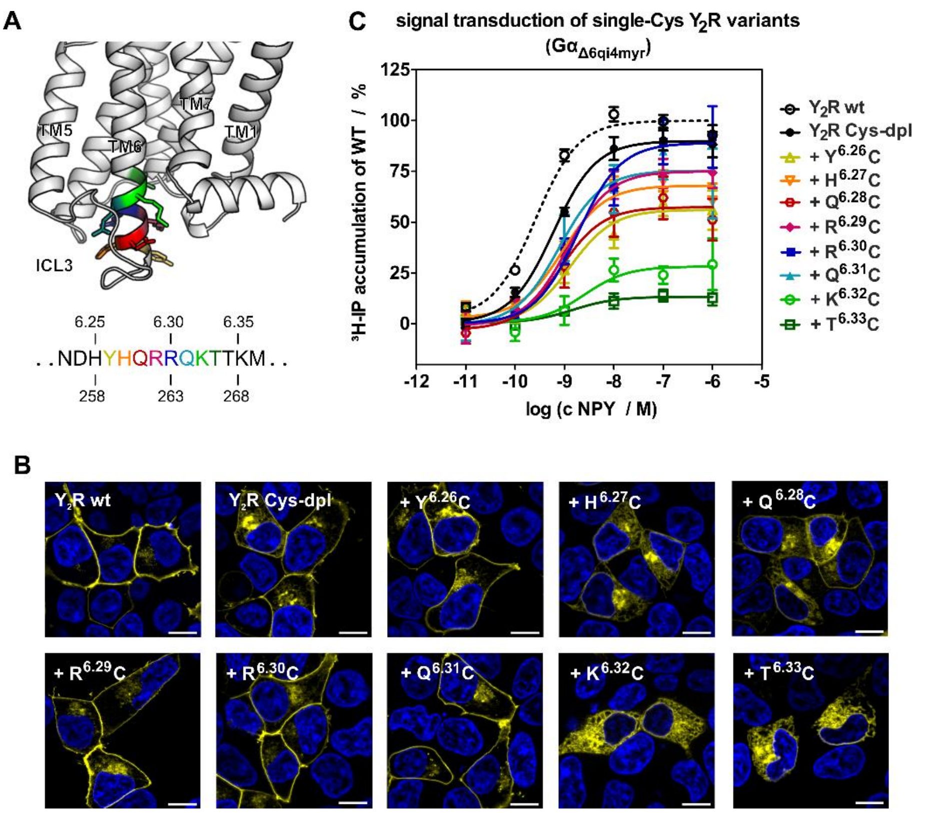

Figure 2S: Positional scan to identify suitable positions for cysteine introduction. (A) Eight consecutive residues at the intracellular end of transmembrane helix 6 (TM6) were exchanged to cysteine in the cysteine-depleted Y_2R base mutant (Y_2R Cys-dpl). Positions are indicated on Y_2R model from (14). (B) Cell surface localization of eYFP fusion proteins (depicted in yellow) was investigated in transiently transfected HEK293 cells. $R^{6.29}C$, $R^{6.30}C$ and $Q^{6.31}C$ displayed best surface localization, while $K^{7.32}C$ and $T^{6.33}C$ appeared to be mostly trapped intracellularly. Nuclei were stained with Hoechst33342 and depicted in blue, bar equals 10 µm. (C) Y_2R variants were also tested for signal transduction in transiently transfected COS7 cells via a chimeric $G\alpha_{\Delta 6qi4myr}$ protein. Again, cysteine introduction in positions 6.29-6.31 produced best signal.

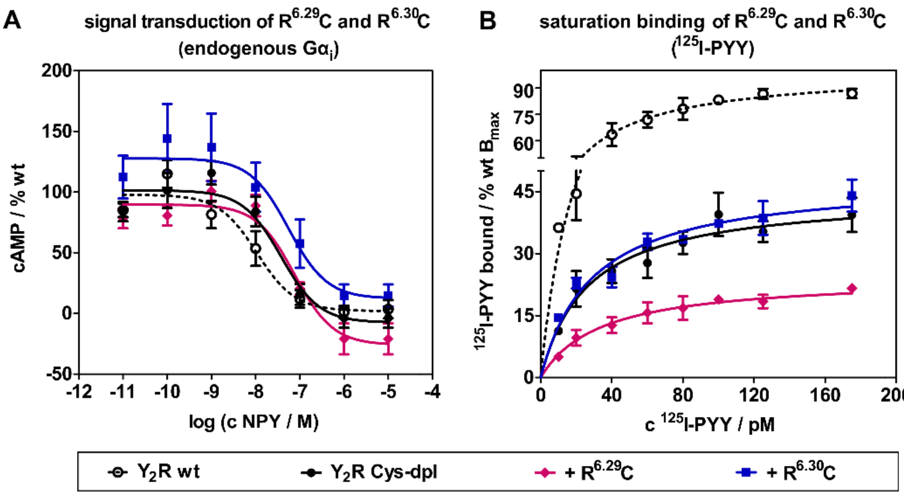

Figure S3: Detailed characterization of Y2R Cys-dpl + R6.29C/R6.30C. (A) Y2R variants were tested in a cAMP-based signal transduction assay using the endogenous $G\alpha i$ protein to ensure wild type-like behavior. (B) Saturation binding assays with ^{125}I-PYY revealed reduced expression,

but ligand affinity remained unchanged.

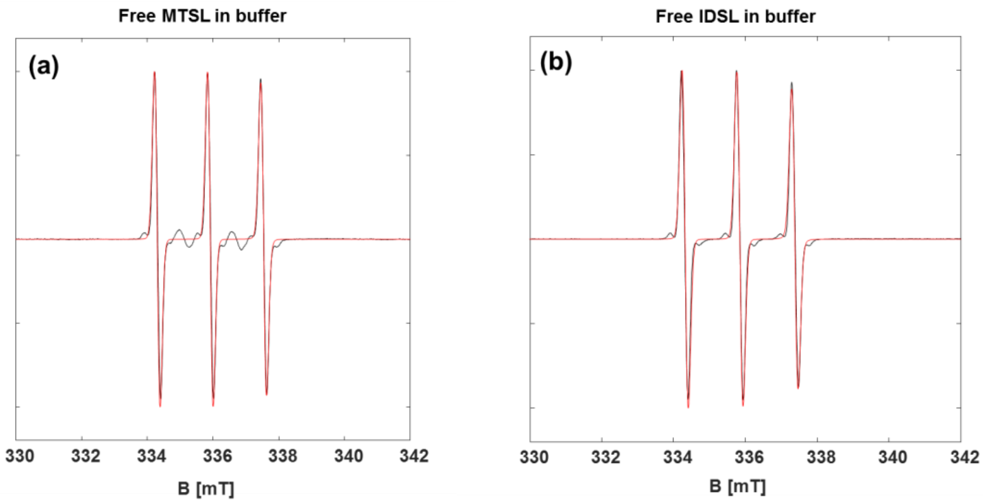

Figure S4: Room temperature X-band EPR spectra of the used spin labels in NaP buffer (a) Experimental (black) and simulated (red) CW-EPR spectra of free MTSL and (b) free IDSL after treatment with equimolar DTT in buffer. Simulations revealed a more pronounced hydrophobic nature of IDSL, possessing an A_{iso} of 42.9 MHz compared to MTSL (A_{iso}=45.4 MHz).

29

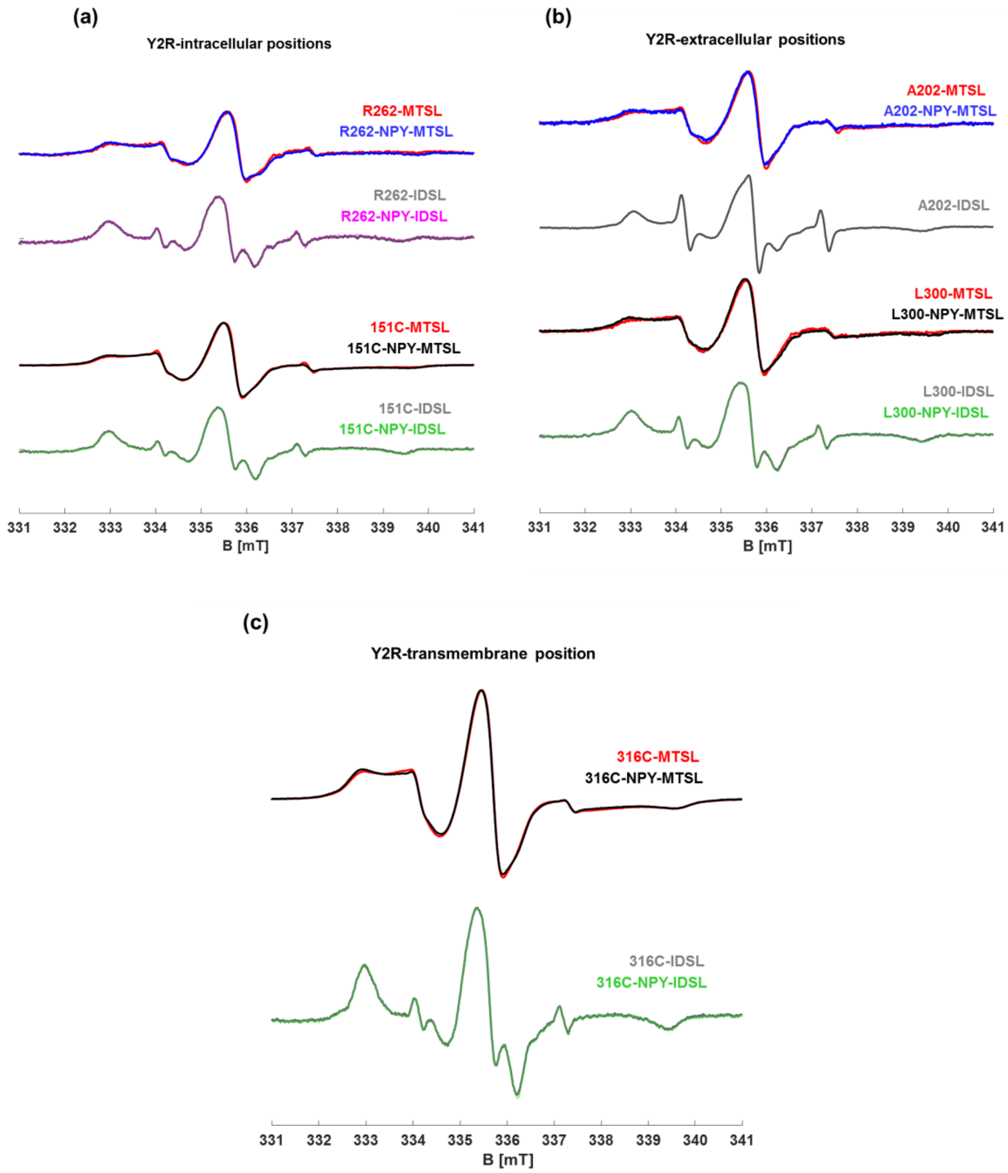

Figure S5 (a-c): Room temperature X-band EPR spectra of the single mutants labeled with MTSL and IDSL in the presence and absence of NPY ligand for (a) intracellular, (b) extracellular and (c) transmembrane positions.

30

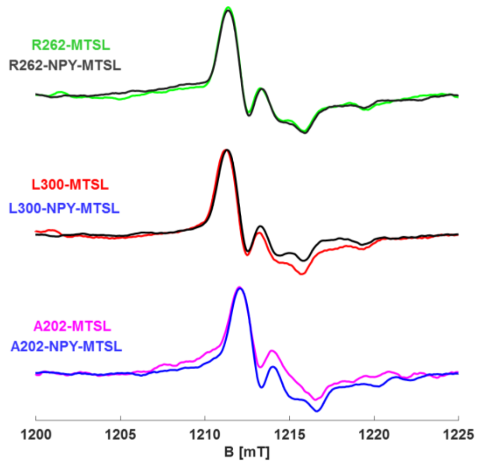

Figure S6: Overlaid low temperature (100K) Q-band CW-EPR spectra of the two extracellular and transmembrane positions and labeled with MTSL in the presence and absence of the NPY ligand

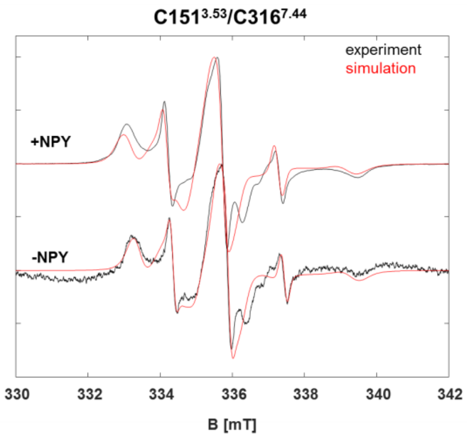

Figure S7: Room temperature CW-EPR experimental (black) and simulated (red) spectra of double mutants, labeled with IDSL. With and without the NPY ligand.

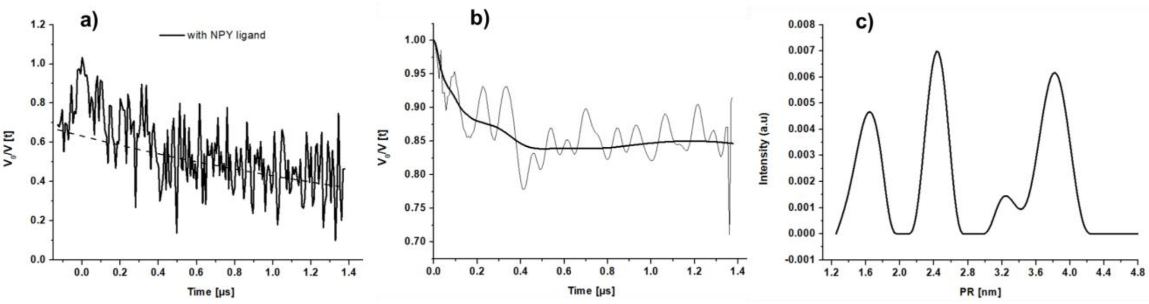

Figure S8: DEER measurements of the Y2 receptor at different offsets in the presence of NPY at 40MHz. From left to right are given DEER raw data (left) with applied background lines (dashed), background corrected DEER time traces with their fit(solid line) (in center) and the corresponding distance distributions

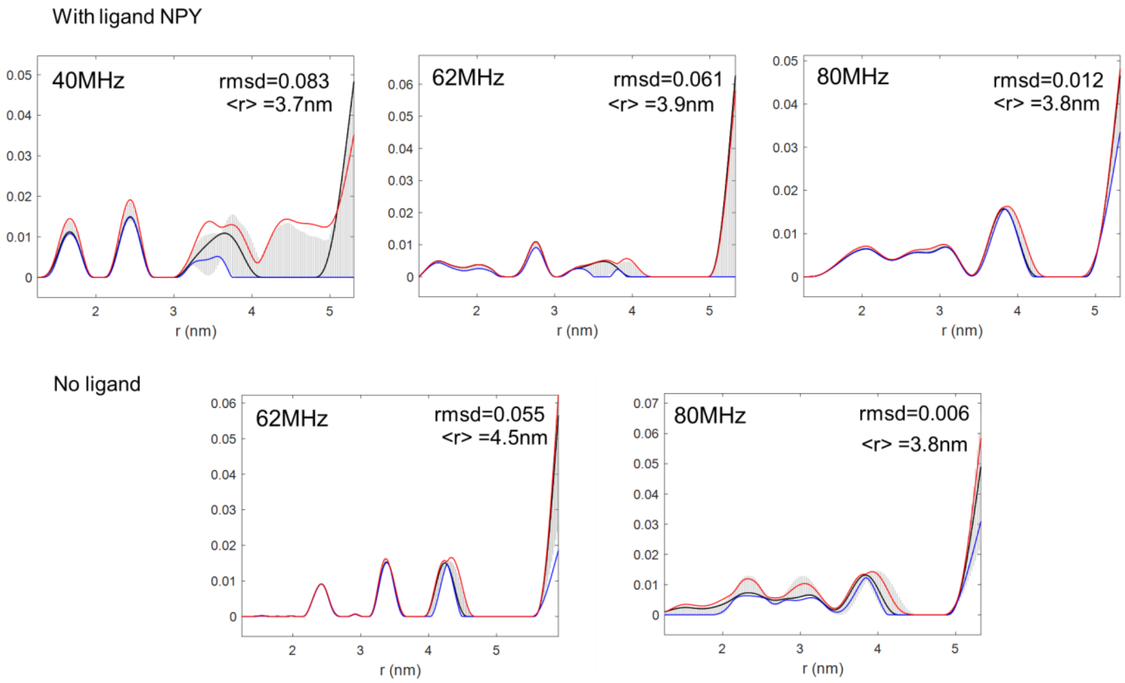

Figure S9: The influence of uncertainties of the background correction and dimensionality on the distance distributions in the presence and absence of NPY ligand at different offsets. The error analysis is based on validation tool implemented in DEER analysis. A total of 231 trials were used for each offset. The background corrections were varied between 800 and 1000 ns (21 trials) and dimensionality was changed between 2.0 to 3.0 (11 trials). The rmsd of the best fit and the mean distances, <r>, for each offset are given as insets.
